# Intermediate filaments spatially organize intracellular nanostructures to produce the bright structural blue of ribbontail stingrays across ontogeny

**DOI:** 10.3389/fcell.2024.1393237

**Published:** 2024-07-10

**Authors:** Michael J. Blumer, Venkata A. Surapaneni, Jana Ciecierska-Holmes, Stefan Redl, Elisabeth J. Pechriggl, Frederik H. Mollen, Mason N. Dean

**Affiliations:** ^1^ Institute of Clinical and Functional Anatomy, Medical University Innsbruck, Innsbruck, Austria; ^2^ Department of Infectious Diseases and Public Health, City University of Hong Kong, Kowloon, Hong Kong SAR, China; ^3^ Department of Biomaterials, Max Planck Institute of Colloids and Interfaces, Potsdam, Germany; ^4^ Institute of Neuroanatomy, Medical University Innsbruck, Innsbruck, Austria; ^5^ Elasmobranch Research, Bonheiden, Belgium

**Keywords:** elasmobranchs, skin, chromatophore unit, iridophores, vesicles, guanine, melanophore, mosaic organelle

## Abstract

In animals, pigments but also nanostructures determine skin coloration, and many shades are produced by combining both mechanisms. Recently, we discovered a new mechanism for blue coloration in the ribbontail stingray *Taeniura lymma*, a species with electric blue spots on its yellow-brown skin. Here, we characterize finescale differences in cell composition and architecture distinguishing blue from non-blue regions, the first description of elasmobranch chromatophores and the nanostructures responsible for the stingray’s novel structural blue, contrasting with other known mechanisms for making nature’s rarest color. In blue regions, the upper dermis comprised a layer of chromatophore units —iridophores and melanophores entwined in compact clusters framed by collagen bundles— this structural stability perhaps the root of the skin color’s robustness. Stingray iridophores were notably different from other vertebrate light-reflecting cells in having numerous fingerlike processes, which surrounded nearby melanophores like fists clenching a black stone. Iridophores contained spherical iridosomes enclosing guanine nanocrystals, suspended in a 3D quasi-order, linked by a cytoskeleton of intermediate filaments. We argue that intermediate filaments form a structural scaffold with a distinct optical role, providing the iridosome spacing critical to produce the blue color. In contrast, black-pigmented melanosomes within melanophores showed space-efficient packing, consistent with their hypothesized role as broadband-absorbers for enhancing blue color saturation. The chromatophore layer’s ultrastructure was similar in juvenile and adult animals, indicating that skin color and perhaps its ecological role are likely consistent through ontogeny. In non-blue areas, iridophores were replaced by pale cells, resembling iridophores in some morphological and nanoscale features, but lacking guanine crystals, suggesting that the cell types arise from a common progenitor cell. The particular cellular associations and structural interactions we demonstrate in stingray skin suggest that pigment cells induce differentiation in the progenitor cells of iridophores, and that some features driving color production may be shared with bony fishes, although the lineages diverged hundreds of millions of years ago and the iridophores themselves differ drastically.

## Introduction

Animal coloration serves a variety of functions, including communication, camouflage from predators, and thermoregulation (Sun et al., 2013). In most cases, skin color is due to the presence of specialized cells, collectively referred to as chromatophores, that produce a wide range of natural hues (Bagnara et al., 1968; Stavenga, 2014). Pigment-containing chromatophores include melanophores (black-brown pigment), xanthophores (yellow pigment), and erythrophores (red pigment), which selectively absorb some wavelengths of light while reflecting others, thereby producing colors that do not change at any observation angle. On the other hand, chromatophores called iridophores lack pigments and instead use nanostructural architectures (e.g., guanine or isoxanthopterin crystals) to selectively reflect light (Ligon and McCartney, 2016). These so-called ‘structural colors’ can produce strikingly brilliant colors that can be either iridescent or non-iridescent (dependent on or independent of viewing/illumination angle) (Taylor and Bagnara, 1972; Simonis and Berthier, 2012; Figon et al., 2021). Iridophores are not only found in the skin, but occur in other organs, such as the eyes of crustaceans, where reflector cells with isoxanthopterin crystals produce non-iridescent colors (Palmer et al., 2020; Shavit et al., 2023).

In most vertebrates, color-determining cells are located in the dermis and usually involve multiple chromatophore types (Bagnara et al., 1968). Typically, the various chromatophores are by no means randomly distributed, but form several discrete layers, together referred to as the dermal chromatophore unit. Despite variation across species, this stratified unit generally comprises an uppermost single layer of xanthophores or erythrophores, an intermediate layer of crystal-containing iridophores, and finally a layer of branched melanophores whose thin processes extend upwards and interact with the iridophores (Bagnara et al., 1968; Hirata et al., 2003; Bagnara et al., 2007). Dermal chromatophores function as layered filters, keeping pigment- and crystal-containing cells in close association. It is the combined action of pigments and crystals that controls color production, with the first layer absorbing shorter wavelengths of incident light, while longer wavelengths passing the first filter are either scattered and reflected back by the iridophores or absorbed by the melanophores (Taylor and Bagnara, 1972; Ligon and McCartney, 2016). On the other hand, colors can also be generated by extracellular nanostructures that vary in shape and/or composition, fashioned from chitin or keratin scales or rods or from collagen fibrils (Prum and Torres, 2003; Prum and Torres, 2004; Prum et al., 2004; Bagnara et al., 2007; D'Alba et al., 2011; Parnell et al., 2015; Orozco et al., 2017). In this way, nature has produced a variety of natural hues through variations in the structure of dermal chromatophore units (e.g., variations in the presence, density and arrangement of certain chromatophores) or through the development of optical extracellular nanostructures.

Among all hues in the animal kingdom, blue is surprisingly rare, but occurs in almost all classes of vertebrates and in many species of mollusks and arthropods (Bagnara et al., 2007; Mäthger et al., 2009; Hsiung et al., 2015). In these diverse species, the blue color is almost always structural, with cases of true pigments reported in just two genera of butterflies (*Papilio* and *Graphium*) and at least two species of callionymid fish (Vuillaume and Barbier, 1969; Goda and Fujii, 1995; Simonis and Berthier, 2012; Goda et al., 2013). Instead, the ‘structural’ blues of most animals rely on physical mechanisms to produce color, with the nanoscale arrangement, morphology and optical properties of scattering elements in their cells and tissues controlling how light is reflected and transmitted. The mechanisms used are diverse and may include thin film/multi-layer interference, diffraction, and coherent/incoherent scattering, or a combination of these mechanisms. The blue coloration of numerous bony fish (teleosts), for instance, is the result of multilayer interference that occurs in a stack of light-reflecting guanine crystals (Land, 1972; Zhao et al., 2022). In contrast, in mammals or in the feathers and skin of birds, blue color derives from coherent scattering from either photonic-crystal-like arrangement of melanin granules; quasi-ordered air cavities in a keratin matrix; or arrays of densely packed and quasi-ordered collagen fibril bundles present in the deeper dermis (Prum and Torres, 2003; Prum and Torres, 2004). Often, an underlying melanin pigment layer absorbs longer wavelengths to produce brighter blues (Prum and Torres, 2003; Prum and Torres, 2004; Bagnara et al., 2007; Simonis and Berthier, 2012; Sun et al., 2013).

Among fishes, elasmobranchs (sharks and rays) are generally not conspicuously colored and therefore have been of little concern to scientists interested in how nature makes color. Blue coloration, as far as we know, has only been explored in two species, an electric ray (*Torpedo ocellata,* a junior synonym of *T. torpedo;* Linnaeus, 1758) and a ribbontail stingray (*Taeniura lymma*; Fabricius, ex Forsskål, in Niebuhr, 1775), both of which bear prominent blue spots on their backs (Taylor and Bagnara, 1972; Bagnara et al., 2007; Surapaneni et al., 2024). In the electric ray*,* ultrastructural data on the cellular architecture of the skin have never been published, but in a literature review by Bagnara et al. (2007), an artist’s reconstruction shows histological characteristics of the spot and adjacent region, however with no cellular and intracellular details. The blue spots are surrounded by a dark ring, in turn by a concentric pale area, contrasting the spot against the brown dorsal skin. Bagnara et al. (2007) reported that the epidermis of the blue spot contains no melanophores, while cells with “exceedingly large melanosomes” (their exact size not reported) are present in the dermis, embedded in a thick collagen layer that they argue is responsible for the blue hue, not unlike the collagen-based mechanisms known in various birds and mammals (Taylor and Bagnara, 1972; Prum and Torres, 2003; Prum and Torres, 2004; Bagnara et al., 2007).

**FIGURE 1:**
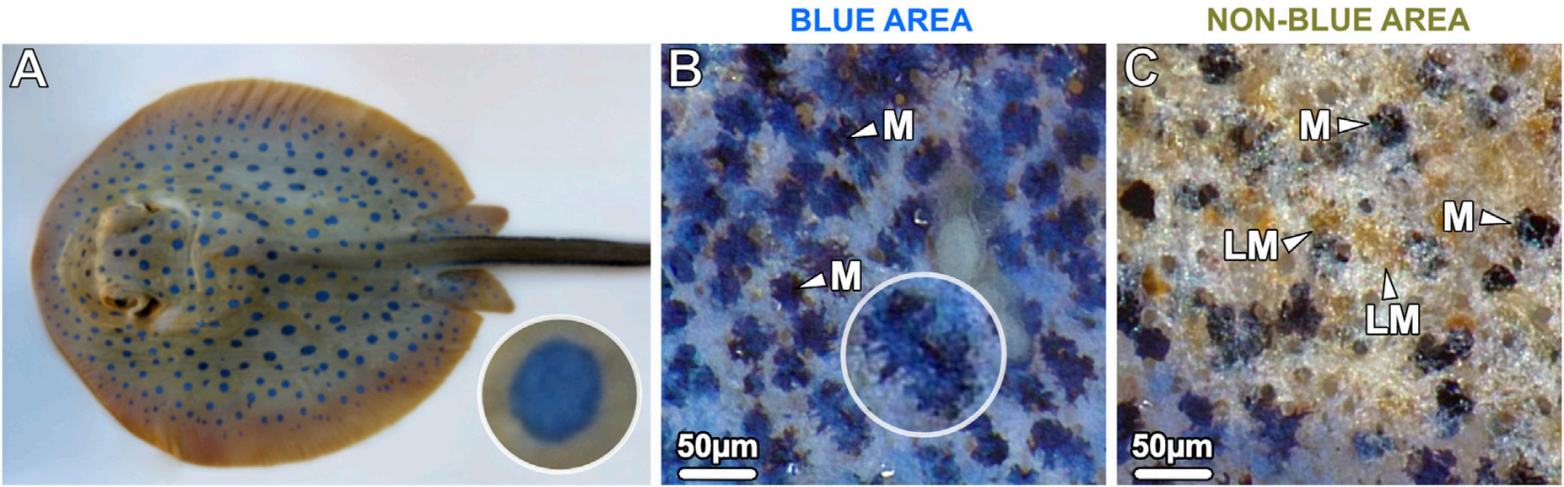
**(A)** The blue spotted ribbontail ray *T. lymma,* **(B, C)** examined with a stereomicroscope at higher magnification. **(B)** The blue areas show a speckled pattern consisting of numerous small blue splotches (40–60 µm in diameter) interspersed with black dendritic melanophores (M) in comparatively high density on a white-bluish background. **(C)** The non-blue areas show aggregated brown (light-pigmented) melanophores (LM) and individual, sparse, roundish black melanophores (M). In the bottom left of the image the blue area is visible.

In contrast, we have recently shown that the blue color (reflectance peaks ∼447–452 nm) of the ribbontail stingray (*T. lymma*; Figure 1A) is due to a novel type of iridophore that occurs in the epidermis and in exceptionally high density in the dermis, equipped with a stable colloidal system of crystal-containing vesicles (referred to as iridosomes in the present study) that coherently scatter incident light, while associated melanophores absorb the longer incident wavelengths (Surapaneni et al., 2024). Surapaneni et al. (2024) is the first study to describe structural color formation in elasmobranchs, using a combination of optical, histological and modeling methods to explain the basis for the non-iridescent blue of the ribbontail ray.

In the current study, our goal is to provide a detailed ultrastructural description of the ribbontail ray’s novel iridophore, using finescale morphological characterizations (e.g., scanning and transmission electron microscopy) to investigate how the colloidal system of iridosomes is stabilized intracellularly, and provide evidence that this cell type can migrate from the dermis into the epidermis. In addition, we demonstrate the presence of mixed pigment organelles (containing both pigments and crystals) in the blue regions and highlight the differences in cell composition and nanoscale architecture that distinguish blue from non-blue regions, thereby framing tissue alterations that decide body pattern and supported the evolution within elasmobranchs of nature’s rarest color. By contrasting the cellular anatomies and ultrastructures in blue and non-blue regions of skin, we provide a natural test of the optical morphology hypothesis posed by Surapaneni et al. (2024) that cellular components and their nanoscale arrangements differ in characteristic, hierarchical and predictable ways between the two tissue regions.

## Materials and methods

### Sample collection

Specimens of the blue-spotted ribbontail ray *T. lymma* were from the same individuals used in Surapaneni et al. (2024), collected opportunistically from commercial fisheries operating off Singapore (Pulan Ubin–Changi area), Indonesia (Jakarta), and Kenya (Eastern Indian Ocean) between May 2017 and June 2022, following the sampling protocol by Mollen (2019). Specimens in this study included two females, one adult of ∼26.0 cm DW (disc width) and 72.5 cm TL (total length) and one juvenile of ∼14.9 cm DW and 35.7 cm TL. Immediately after fish death, skin samples (approximately 8 × 8 × 8 mm) were fixed for either light or electron microscopy (see below). Samples were stored in the relevant fixative (see below) at 4 °C for 1 week and shipped to the Medical University of Innsbruck, where they were further processed. The samples were cut into smaller pieces, each containing a blue spot and adjacent non-blue tissue. Several tissue blocks from the young and adult specimens were examined.

### Light microscopy (LM)

Samples were fixed in 4% paraformaldehyde (PFA) in 0.1 M phosphate buffer saline (PBS, pH = 7.4), rinsed in PBS, and one set of samples was dehydrated in a graded isopropanol and xylene series and embedded in paraffin using a routine histological infiltration processor (Miles Scientific Inc., Naperville, IL, USA). Vertical serial sections (7 µm) were cut on a HM 355 S microtome (Microm, Walldorf, Germany) and stained either with hematoxylin and eosin (H&E) (Shandon Varistain 24–4, Histocom Vienna, Austria) or with periodic acid-Schiff (PAS) reaction according to the manufacturer’s protocol. PAS was used to detect deposits of mucosubstances such as glycoproteins and glycolipids in the sections. A second set of samples was embedded in a cryomatrix (Tissue-Tek O.C.T compound, Science Services, Munich, Germany) after rinsing in PBS and vertical serial frozen sections (50 µm) were cut using a CM3050S cryostat (Leica, Wetzlar, Germany). These sections were not stained. Paraffin, cryo- and semi-thin resin sections (see below) were examined with a Zeiss ax10 microscope equipped with a Zeiss AxioCam 512 color digital camera and ZEN 3.0 blue edition software (Zeiss, Oberkochen, Germany).

### Tissue preparation for transmission (TEM) and scanning electron microscopy (SEM)

Samples were fixed in 2.5% glutaraldehyde (GA) and 2% PFA in 0.1 M sodium cacodylate buffer (pH = 7.4), rinsed in sodium cacodylate buffer and post-fixed in 0.5% osmium tetroxide and 1% potassium ferricyanide in distilled water overnight at 4 °C. Samples were rinsed again, dehydrated in a graded ethanol series and acetone and embedded in EPON resin (#45359, Sigma-Aldrich, Austria). Vertical serial semi-thin sections (1–2 µm) were cut on a Reichert Ultracut S microtome (Leica Microsystem, Wetzlar, Germany) with a Histo-Jumbo diamond knife (Diatome, Biel, Switzerland) and stained with toluidine blue (TB) for 3 min at 60°C. Semi-thin toluidine-blue-stained sections were observed with light microscopy first to guide TEM observations of ultra-thin sections from the same block. Unstained semi-thin were observed with SEM (see below). Vertical serial ultra-thin sections (90 nm) for TEM were cut with an ultradiamond knife (Diatome, Biel, Switzerland), mounted on dioxane formvar-coated slot grids (#PYSL2010S-CU, Science Services, München, Germany), and stained with 1% uranyl acetate and lead citrate (after Reynolds, 1963).

### TEM of phosphotungstic acid-stained ultra-thin sections

A small number of ultra-thin sections were collected on 100 µm copper hexagonal grids, incubated with prewarmed 1% phosphotungstic acid in 95% ethanol for 5 min at 55°C, followed by three 5-min washes in absolute ethanol, and then air-dried. Ultra-thin sections were examined at 80 kV with a Philips CM 120 transmission electron microscope (FEI, Eindhoven, Netherlands) equipped with a MORADA digital camera and iTEM software (Olympus SIS, Münster, Germany).

### Measurements of cellular nanostructures

The iTEM software was used to take the following measurements (n=50 each): size of iridosomes, vesicles and crystals, spacing of iridosomes and vesicles, diameter of intermediate filaments and the size of melanosomes. Statistical comparisons of sizes were made in Python software, using non-parametric Kruskal-Wallis tests with the *scipy* module, with plots generated using the *seaborn* module.

### SEM of semi-thin sections

Unstained semi-thin sections (2 µm) were mounted on glass slides and the superficial layer of resin removed by treating sections with 10% potassium hydroxide (KOH) in absolute methanol for 30–90 s at room temperature. Sections were then rinsed three times in absolute ethanol for 5 min each. The slides were broken and the bottom side attached to SEM pins with carbon adhesive tabs. A conductive connection was then applied between the upper side of the slides and the pin stubs using conductive carbon cement. Sections were sputter coated with 3 nm Au/Pd (Balzers) and examined with a Zeiss DSM 982 Gemini operated at 1–3 kV.

### Literature analysis

In order to understand the morphology of ribbontail stingray scattering elements in a broader functional context, a Factor Analysis of Mixed Data (FAMD) was performed including morphological features from diverse literature examples of natural structural “blues” (388–490 nm). Similar to Principal Component Analysis (PCA) and Multiple Correspondence Analysis (MCA), FAMD reduces the dimensionality of complex datasets, but allows simultaneous analysis of continuous and categorical variables. This can reveal hidden correlations among variables as starting points for more formal downstream hypothesis testing (e.g., pairwise correlation, chi-squared test). Variables included in our FAMD analysis related to the structural and optical properties of scatterers (summarized in Supplementary Table S1) from diverse eukaryotic groups (vertebrates, invertebrates and algae; Prum et al., 1999; Prum and Torres, 2004; Prum et al., 2004; Noh et al., 2010; Saenko et al., 2013; Teyssier et al., 2015; Gur et al., 2015a; Gur et al., 2015b; Gur et al., 2020; Chandler et al., 2015; Hsiung et al., 2015; Lopez-Garcia et al., 2018; Jeon et al., 2023; Surapaneni et al., 2024). FAMD analysis and plotting were performed using *FactoMineR* and *Factoextra* packages in R software version 4.2.2 (Lê et al., 2008; Kassambara and Mundt, 2020; R Development Core Team, 2023). Additionally, the structural and optical properties of various blue-producing structures were plotted using *pandas, matplotlib and seaborn* modules in python software.

## Results

Juvenile and adult specimens of the ribbontail ray had striking electric blue spots (0.5–0.7 cm in diameter) on the yellow-brownish background of their dorsal skin. Despite the uniform appearance of the blue spots at low magnification, examination of the blue spots with a stereomicroscope at higher magnification revealed a speckled appearance consisting of numerous small translucent blue splotches (40–60 µm in diameter) interspersed in a semi-uniform dispersion of black dendritic melanophores on a white-bluish background (Figures 1A, B). In contrast, the adjacent non-blue regions had far more sparse and rounded black melanophores, scattered among aggregated brown melanophores (Figure 1C). To investigate the region-specific differences at the cellular and intracellular level, vertical sections were examined by light and electron microscopy, respectively.

### The epidermis

**FIGURE 2:**
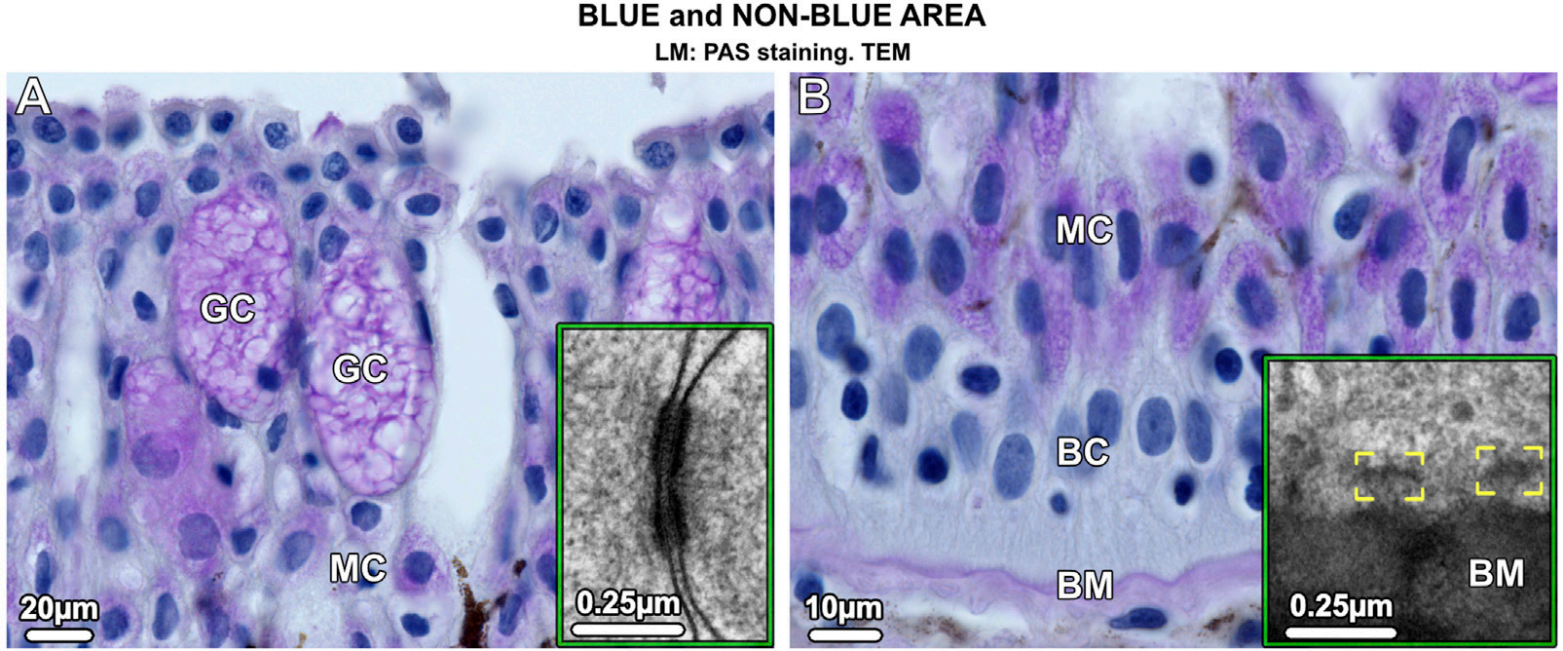
Vertical sections of the multilayered epidermis of ribbontail stingray, showing mucus-containing cells and basal cells in the **(A)** upper and **(B)** lower epidermis. **(A)** Goblet cells (GC) and **(B)** mucocytes (MC) stain purple after PAS staining. In addition, the basement membrane (BM) stains purple, but no staining is visible in the basal cells (BC) that form the deepest layer of the epidermis. Desmosomes are abundant in the epidermis connecting neighboring cells (inset in **A**). The basal cells (BC) are anchored to the basement membrane (BM) via hemidesmosomes (brackets, inset in B).

In blue and non-blue areas, the epidermis was histologically similar and consisted of a multilayered epithelium (up to 250 µm thick in adults) dominated by two types of mucus-containing PAS (periodic acid-Schiff)-positive cells: large goblet cells (50–70 µm in size) and small columnar mucocytes (20 µm in size) (Figures 2A, B). Desmosomes were abundant connecting both types of mucus cells and basal cells, the latter forming the deepest epidermis and attached to the underlying basement membrane by hemidesmosomes (Figures 2A, B insets). The epidermis, however, differed in several aspects that distinguished the blue from the non-blue regions.

**FIGURE 3:**
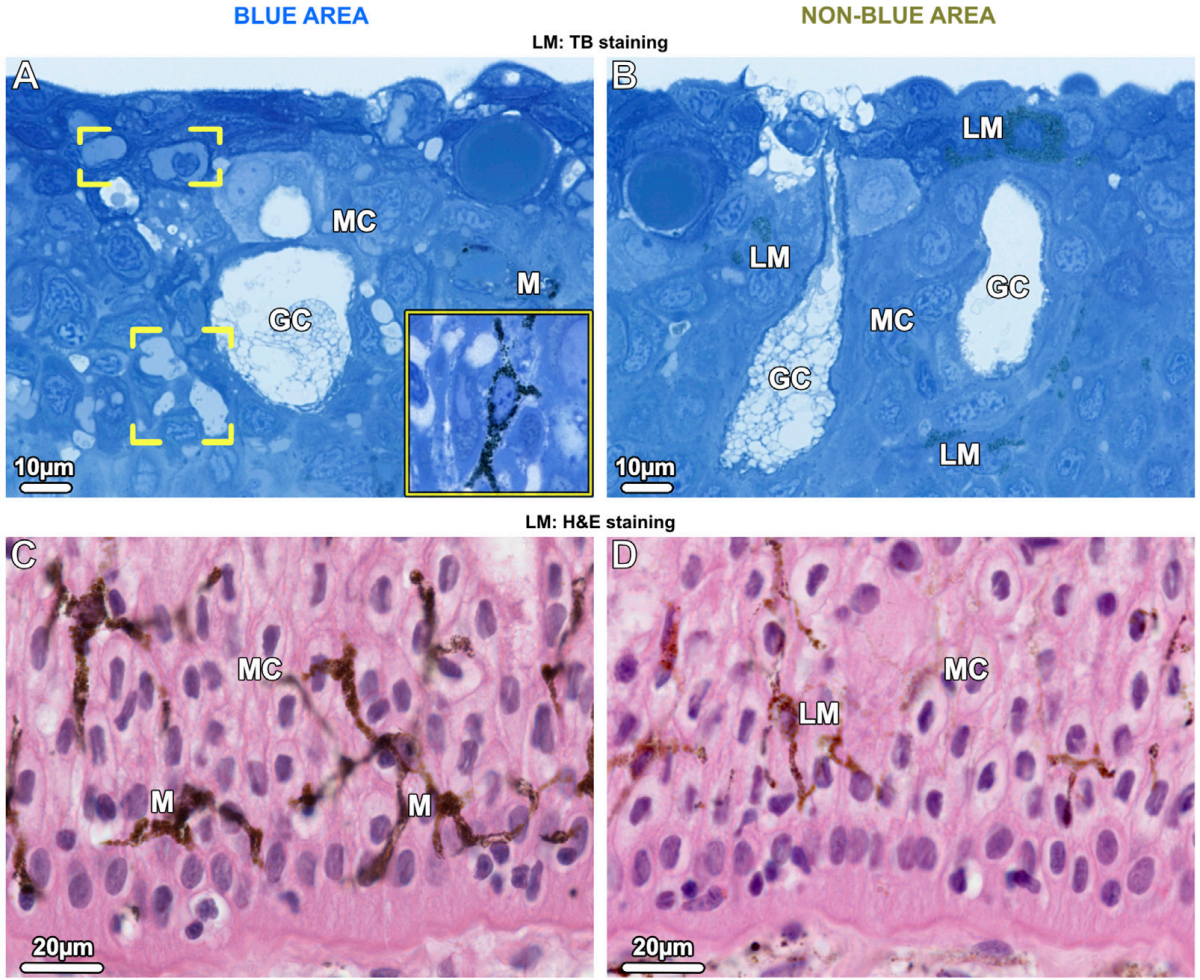
LM Vertical sections of the epidermis show chromatophore types in blue areas (left images) and non-blue areas (right images). **(A, B)** Upper epidermis. **(A)** In the blue area, a few branched iridophores (brackets) and dark-pigmented (black) stellate melanophores (M and inset) are located among the mucocytes (MC) and goblet cells (GC). **(B)** In the non-blue area, iridophores are absent and light-pigmented (brown) melanophores (LM) are scattered among mucus-containing cells. **(C, D)** In the lower epidermis in both blue and non-blue areas, melanophores are more numerous than in the upper epidermis. In the blue area, **(C)** dark-pigmented (black) melanophores (M) are located among the mucocytes (MC). Although few branched iridophores are also present in this region, it is not possible to distinguish between them and mucocytes (MC) in paraffin sections. **(D)** In the non-blue area, only sparse light-pigmented melanophores (LM) are present.

In the epidermis of the blue areas of both juvenile and adult specimens, iridophores were scattered between goblet cells and mucocytes (Figure 3A; Figure 7A). *Taeniura lymma* iridophores, first described by Surapaneni et al. (2024), have a distinct translucent appearance, especially in electron microscopy, exhibiting fingerlike processes off the cell body and an internal array of quasi-ordered nanovesicles (see below). The processes of iridophores were observed to extend down into the basal layer, some coming in contact with the basement membrane (Figure 7B). Iridophores were a major component of the dermis and are described in more detail in ‘The dermis’ section below. Black melanophores were scattered throughout the epidermis, sparsely in the juvenile, but more abundantly in adult specimens, particularly in the lower epidermis (Figure 3C). In histological cross sections, melanophores had a stellate appearance with long processes extending from the cell body (similar to cellular gross morphologies in stereomicroscopy; Figure 1B) and contained melanosomes evenly distributed throughout the cell (Figure 3A inset). Iridophores and melanophores were never observed to contact each other in the epidermis, but were surrounded by mucus-containing cells, albeit not linked to them via desmosomes.

Iridophores were absent in the epidermis of the non-blue areas of both juvenile and adult specimens. As in blue areas, the melanophores had a stellate shape, however, they were present in far lower density than in the blue regions and were predominantly brown melanophores, their melanosomes staining more lightly in TB- and H&E-stained sections (Figures 3B, D).

### The dermis

**FIGURE 4:**
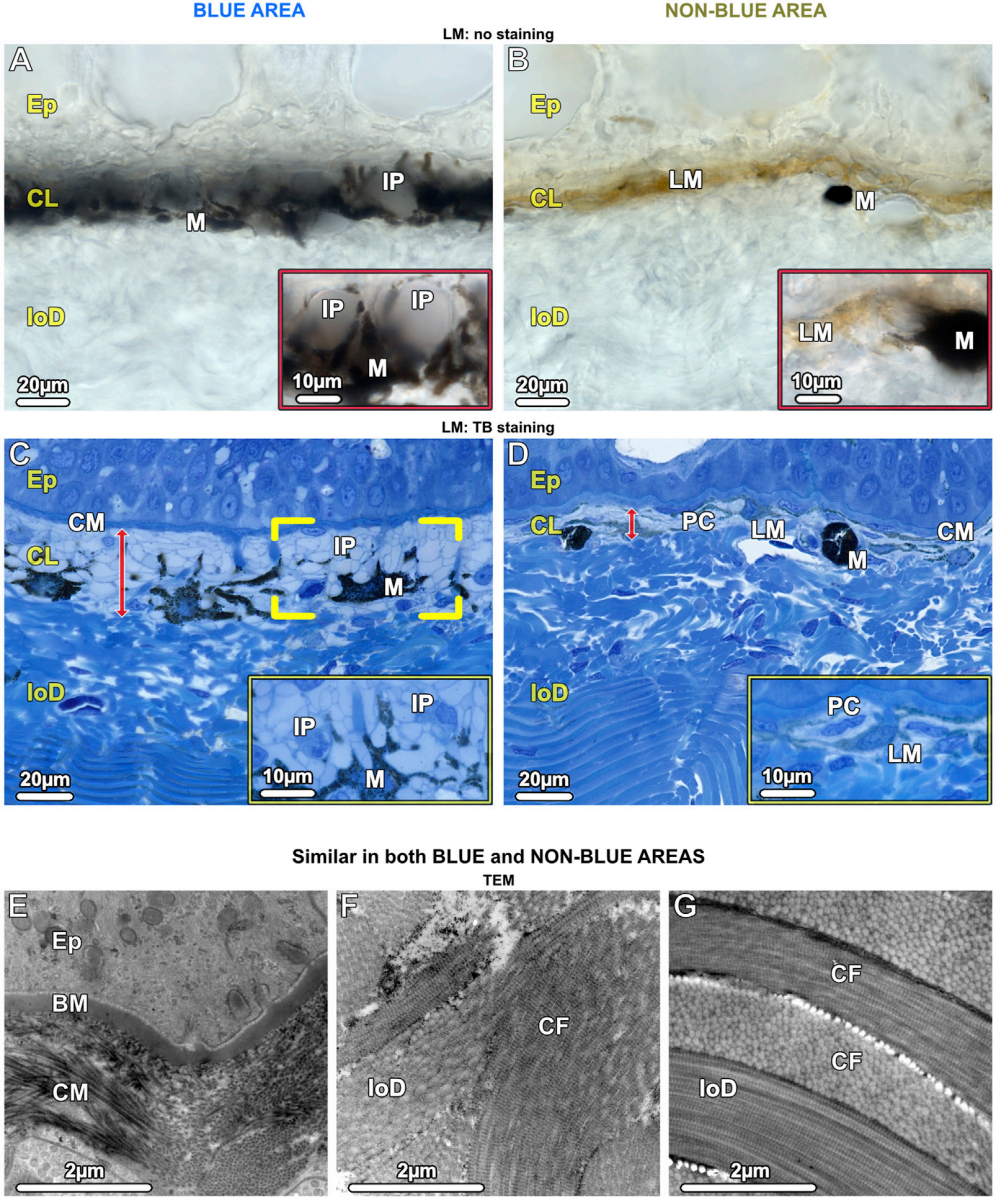
LM **(A–D)** and TEM **(E–G)**. Vertical sections of the chromatophore layer (CL) of the upper dermis and the fibrous tissue of the lower dermis (loD) in both blue (left) and non-blue (right) areas. **(A, B)** Unstained cryosections (50 µm) show the general composition of the chromatophore layer, reflecting differences between blue and non-blue regions seen in the stereomicroscope. **(A)** Translucent iridophores (IP and inset) and black branched melanophores (M) are present in the blue area, while **(B)** predominantly brown (LM) and isolated black melanophores (M) are present in the non-blue area, but no iridophores (inset). **(C, D)** Semi-thin sections (1 µm) show the cellular architecture and thickness (red arrows) of the chromatophore layer (CL), sandwiched between a mat of collagen (CM) and the lower dermis (loD) in both blue and non-blue regions. In the blue area, **(C)** the iridophores (IP) encase a single melanophore (brackets) with several broad processes (inset). In the non-blue area, **(D)** the chromatophore layer is only one-third as thick as in the blue area. It comprises pale cells (PC), showing a different morphology compared to iridophores (inset), and brown, light-pigmented melanophores (LM). The black, dark-pigmented melanophores (M) are sparsely located immediately below the pale cells and light-pigmented melanophores. **(E–G)** In both the blue and non-blue areas, **(E)** the mat of collagen (CM) is composed of irregularly arranged collagen fibrils in contact with the basement membrane (BM) of the epidermis (Ep). **(F, G)** The lower dermis (loD) is divided into two distinct regions that differ in structure: immediately below the chromatophore layer **(F)**, the collagen bundles (CF) are arranged randomly, while in the deepest region of the dermis, **(G)** they are arranged orthogonally (see also **C, D**).

In both blue and non-blue regions, the dermis was multi-layered, involving an upper layer of chromatophores, and a lower dense fibrous tissue layer. Depending on location (blue or non-blue regions), the chromatophore layer consisted of multiple chromatophore types, including iridophores, melanophores, and novel cells we define as ‘pale cells’ (iridophore-like cells, but lacking key features) (Figures 4A–D), which exhibited striking differences in their cellular and subcellular architecture that distinguished the blue from the non-blue regions (see below). The chromatophore layer was sandwiched between an upper meshwork (mat) of collagen fibrils (≤5 µm thick, beneath the epidermal basement membrane) and the lower dermis (Figures 4C–G). The lower dermis could also be divided into two dominant layers, an upper region beneath the chromatophore layer with randomly arranged collagen bundles, and a lower region where collagen bundles were arranged orthogonally (Figures 4C,D,F,G).

In contrast to the lower dermis, the structure of the chromatophore layer varied considerably between blue and non-blue areas (Figures 4C, D). In the blue regions of both juvenile and adult specimens, the chromatophore layer was 30–35 µm thick and consisted of dendritic black melanophores and iridophores in a dense, epithelial-like arrangement, but lacking desmosomes between cells and with negligible extracellular matrix (except for the periodic vertical collagen bundles; see below) (Figure 4C; Figure 7C).

**FIGURE 5:**
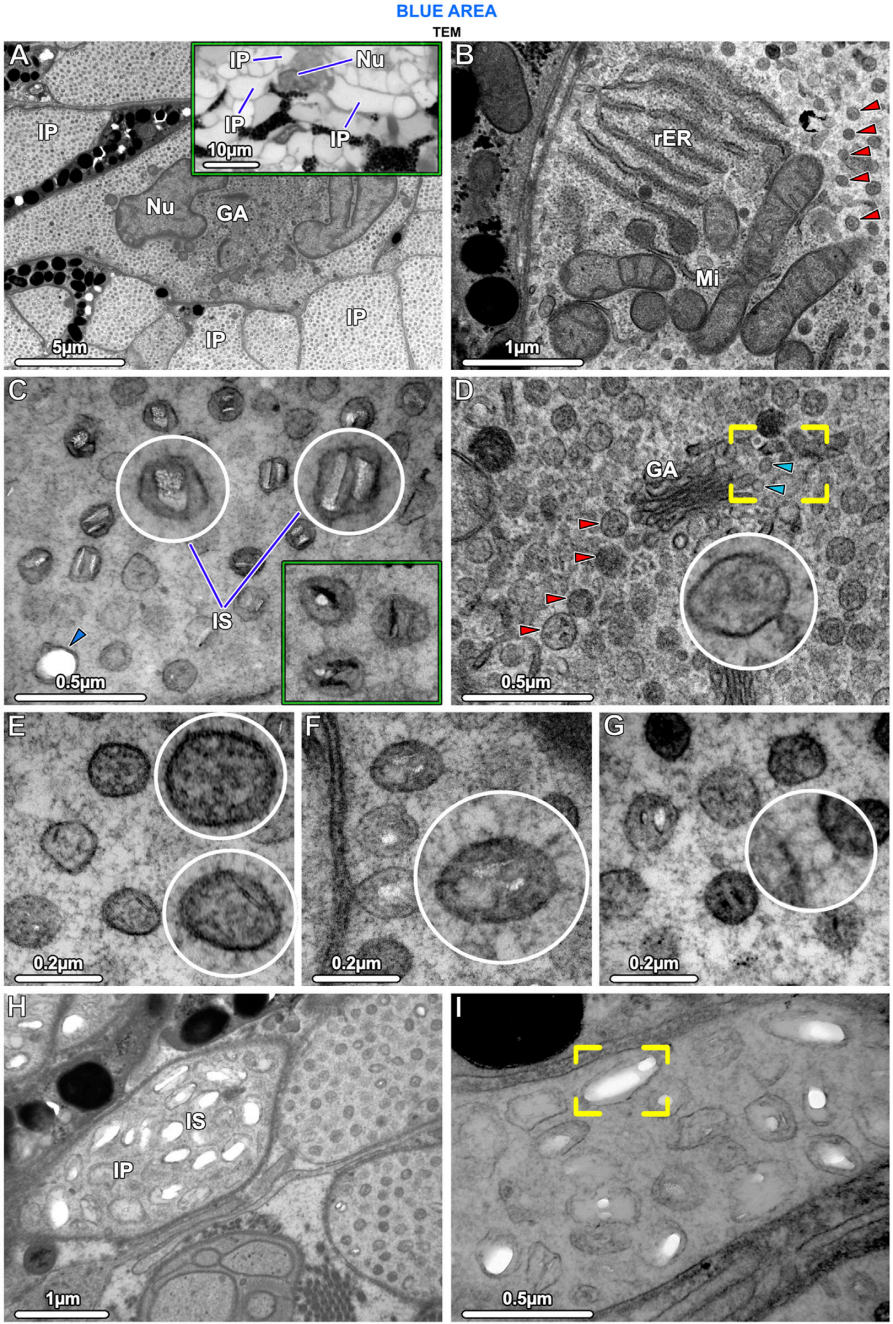
TEM and LM **(**inset in **A)** Ultrastructural characteristics of stingray iridophores. **(A)** The iridophores contain a lobed nucleus (Nu) and several cisternae of Golgi apparatus (GA) and are otherwise packed with iridosomes. Note that the uniform speckling in this image represents the huge numbers of iridosomes within the iridophore and the multiple iridophore processes (IP). The inset shows three processes projecting from an iridophore cell body. **(B)** The cells also contain several mitochondria (Mi) and rough endoplasmic reticulum (rER); the remaining cytoplasm is filled with spherical iridosomes (red arrowheads). **(C)** The iridosomes typically contain two parallel crystal platelets or occasionally a single crystal block, which turn dark (electron-dense) after staining with phosphotungstic acid (rectangular inset; same magnification as **E–G)**. The arrowhead points to an iridosome with an empty oval space. **(D)** Iridosomes (red arrowheads) near the Golgi apparatus are typically densely arranged, surrounded by small vesicles, and apparently containing only fibrillar material. The vesicles (blue arrowheads) appear to have budded off from the flattened Golgi membranes (brackets) to fuse with iridosomes (inset). **(E)** Some iridosomes do not show clear crystals, but contain fibrils sectioned in transverse and longitudinal directions. **(F, G)** The iridophore cytoskeleton consists of a dense network of filaments that **(F)** are radially-arranged around and **(G)** connecting iridosomes. **(H, I)** A few iridophore processes contain larger iridosomes, typically with elongated internal gaps, indicating that their crystals have been extracted during sample preparation (brackets in **I**).

**FIGURE 6:**
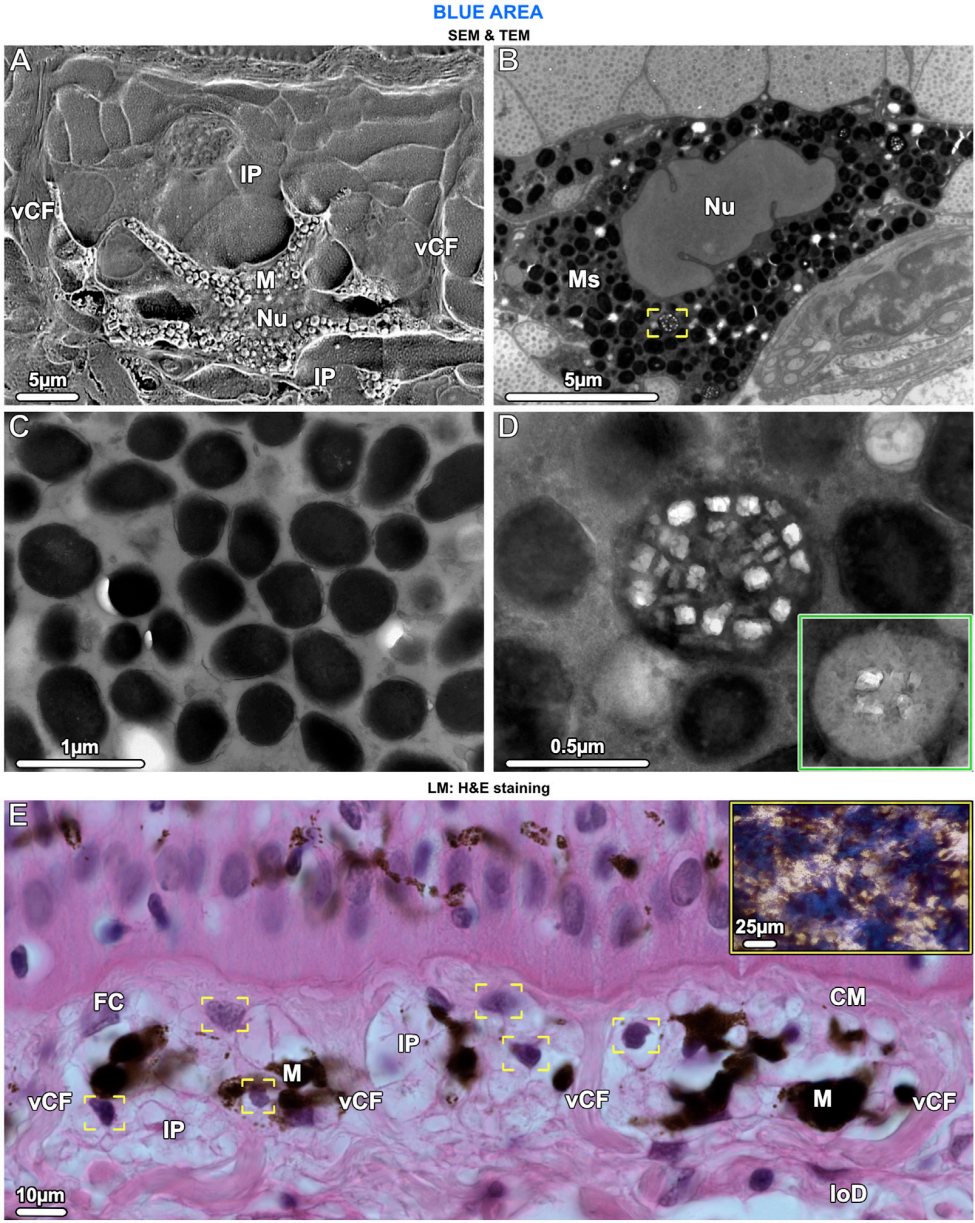
SEM **(A)**, TEM **(B–D)** and LM **(E)**. Ultrastructural characteristics of melanophores and the chromatophore units they form with iridophores. **(A)** The cell body of melanophores (M), which contains the nucleus (Nu), is located in the lower part of the chromatophore layer. Several horizontal and vertical processes originate from the cell body and numerous iridophore processes (IP) envelop the melanophore. Note the vertical collagen fiber bundles (vCF) bounding this chromatophore unit. **(B, C)** Melanophores are densely packed with melanosomes (Ms), which are fully melanized (i.e., completely filled with radio-opaque material), but rarely, some have crystal inclusions (brackets in B, also shown in D at higher magnification). **(D)** When present, the crystals are rectangular and do not turn dark after staining with phosphotungstic acid (as with iridosome crystals), whereas the melanin becomes lighter (inset). **(E)** In the chromatophore layer, each melanophore (M) is surrounded by at least three iridophores (IP), visible by their nuclei (brackets). Chromatophore units are separated from neighboring units by vertical collagen bundles (vCF), connecting the collagen mat (CM) of the upper dermis with the lower dermis (loD). Fibrocytes (FC) are located between the collagen bundles and, in contrast to iridophores, have flat nuclei. The tight association of melanophores and multiple iridophores reflects the combination of blue splotches and stellate melanophores seen in the stereomicroscope (inset and Figure 1B).

**FIGURE 7:**
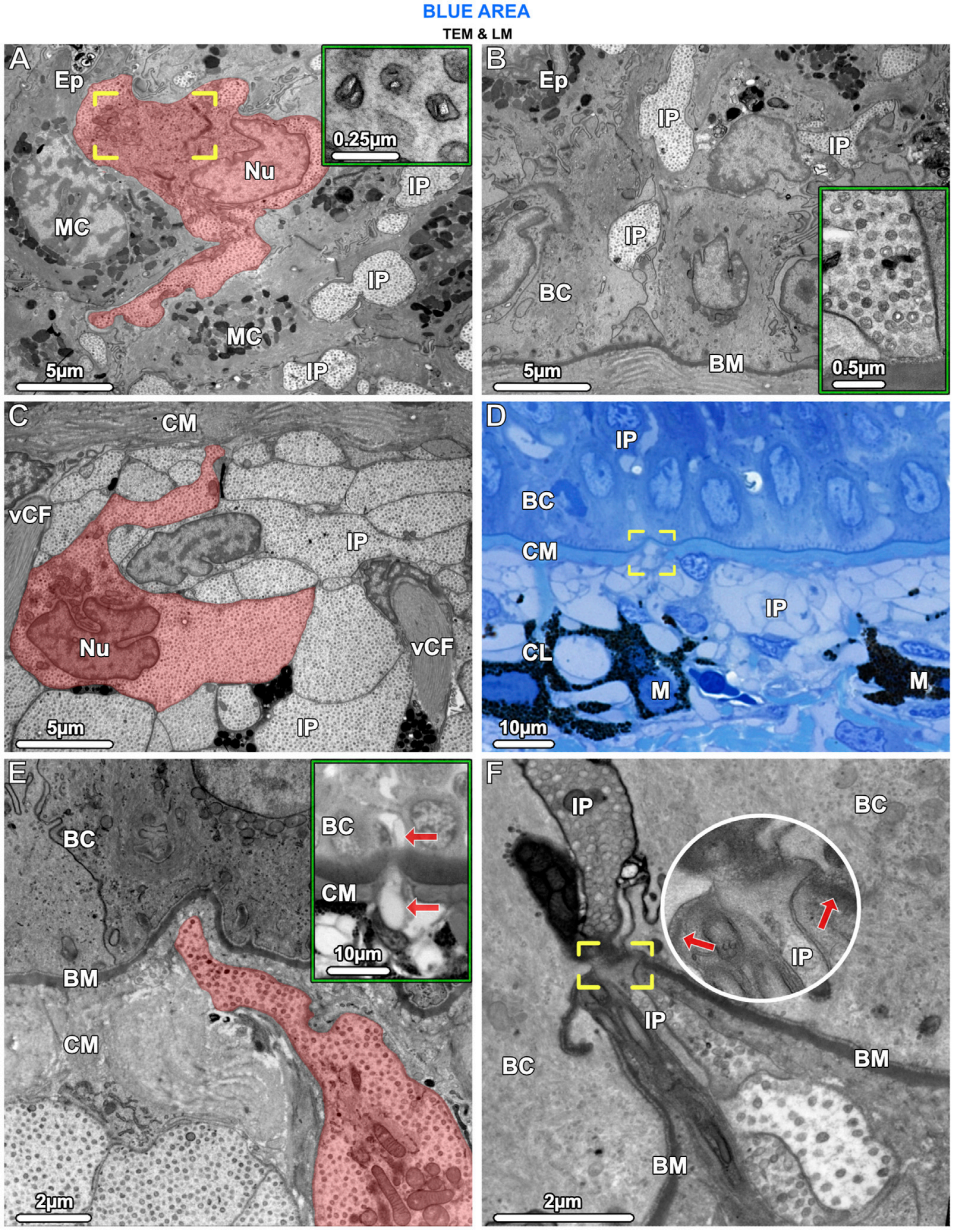
TEM **(A–C, E, F)** and LM **(D)** and inset in **(E)**. Iridophores have the same ultrastructural characteristics in both the epidermis (Ep) and upper dermis and appear to migrate from the chromatophore layer (CL) into the epidermis. **(A)** In the epidermis, an iridophore (highlighted in red) and iridophore processes (IP) are wedged between mucocytes (MC). The iridophore’s lobed nucleus (Nu), crystal-bearing iridosomes (inset) and its Golgi apparatus, surrounded by vesicles (brackets), are visible. **(B)** Iridophore processes (IP) extended into the lowest layers of the epidermis, squeezed between the basal cells (BC). An iridophore process is in contact with the basement membrane (BM) but neither shows hemidesmosome formation there, nor desmosome formation with adjacent cells (inset). **(C)** In the upper dermis**,** below the mat of collagen (CM), processes of iridophores (IP) are arranged in tightly packed layers. An iridophore with its nucleus (Nu) is highlighted in red. Vertical collagen bundles (vCF) run between the iridophore processes (IP). **(D, E)** Iridophore processes (brackets in **D** and highlighted red in **E**) could be seen to break through the collagen mat (CM) beneath the basement membrane (BM) and one process (arrows in the inset) has migrated into the epidermis. **(F)** At the site of penetration, the basement membrane (BM) is interrupted (brackets and arrows pointing to the basement membrane) and the iridophore process (IP) and its iridosome contents are squeezed between the basal cells (BC).

Ribbontail stingray iridophores were remarkable in terms of both shape and ultrastructural organization and strikingly different from iridophores of other vertebrate taxa. The iridophores showed numerous broad and long processes (≤10 µm wide and up to 30 µm long), appearing with varying thicknesses and shapes in our vertical sections (Figure 4C; Figure 5A; Figures 7C, D). These structures were verified as cellular processes rather than individual iridophores by observing them across contiguous serial sections (of 90nm, 1µm and 7µm in thickness) and confirming their lack of nuclei and their attachments to iridophore cell bodies. From individual 2D sections it was impossible to estimate the number of processes per cell, but from the synthesis of all slices we examined and by tracking individual processes across multiple serial sections, we estimate that individual iridophores likely bear >10 extensions off their cell bodies. The finger-like processes of iridophores encircled nearby melanophores, with each iridophore associated with a single pigment cell. The iridophore processes encased melanophores completely, being particularly dense above the melanophore in the upper chromatophore layer, creating a near mono-layer just beneath the mat of collagen fibrils (Figure 4C; Figure 6A; Figure 7C). In contrast, the processes formed only a thin layer below the cell bodies of melanophores (Figure 4C; Figure 6A). Thick (7 µm) paraffin sections showed that the two chromatophore types were grouped together into structural units (up to 60 µm in diameter) separated by vertical collagen bundles, with at least three iridophores surrounding a single melanophore (Figure 6E). These chromatophore units correspond to the numerous small blue splotches interspersed with black dendritic melanophores observed by stereomicroscope (Figure 6E inset).

Iridophores contained a multi-lobed nucleus, several mitochondria, and sparse rough endoplasmic reticulum (Figures 5A, B). Several Golgi apparatuses were located in close proximity to the nucleus, a feature we found to be characteristic of all ribbontail ray iridophores (Figure 5A). Iridophores were densely packed with membrane-bound spherical iridosomes (Figures 5A–D), 126.79 ± 13.08 nm in size and separated from their nearest neighbors by 61.13 ± 13.89 nm (Figure 10A). From the TEM data, iridosomes usually contained two platelet-shaped crystals or occasionally a single crystal block (up to 85 nm wide), but a few iridosomes contained only loose fibrillar material (Figures 5C, E). The crystals were separated from the surrounding membrane by a gap of ∼20 nm and stained dark (electron dense) after treatment with phosphotungstic acid (Figure 5C). Some iridosomes exhibited an empty oval or rectangular space (≤90 nm wide; see Figure 5C bottom left) indicating that their crystals had been extracted during sample preparation, a well-known artifact described by other authors (Taylor, 1969; Wagner et al., 2022).

In iridophores, thin filaments (8–10 nm diameter) were observed, arranged radially around iridosomes, connecting them in a dense filamentous network that traversed the entire iridophore cytoplasm (Fig. F, G). We never observed neighboring iridosomes fusing or coalescing. Clusters of iridosomes near to the Golgi apparatus tended to have exclusively fibrillar contents and were surrounded by smaller vesicles merging with them, the vesicles appearing to originate from the nearby Golgi apparatus (Figure 5D). In a few cases, we observed iridophore processes with far larger iridosomes (∼300 nm wide), below the cell bodies of melanophores. These iridosomes had round to ellipsoidal shapes and typically were empty, indicating their crystals had been extracted (Figures 5H, I).

In contrast to the iridophores (predominantly in the upper part of the chromatophore layer), the melanophores were only in the lower part of the chromatophore layer, embedded periodically and with numerous irregularly shaped processes (≤25 µm long) extending from the melanophore cell bodies, either horizontally or vertically toward the epidermis (Figure 4C and Figure 6A). The melanophores contained densely packed melanosomes with round to elliptical shapes, measuring ≤700 nm in diameter. Most melanosomes were full of black (electron-dense) melanin, but a small number contained both melanin and numerous small rectangular crystals, of a size similar to those seen in iridosomes (up to 120 nm wide) (Figures 6B–D). When stained with phosphotungstic acid, these crystals, however, did not change their appearance (Figure 6D inset). We could not detect a well-developed cytoskeleton in melanophores.

In samples from the young individual, we detected single slender iridophore processes, extending superficially from the chromatophore layer to penetrate the mat of collagen fibrils beneath the epidermis (Figures 7D, E). Examination of serial sections showed that the processes eventually penetrated the basement membrane and advanced into the epidermis (Figures 7E, F). At the site of penetration, the processes became far narrower in their diameter, a bottleneck resulting in tight clustering of the iridosomes within the process (Figure 7F).

**FIGURE 8:**
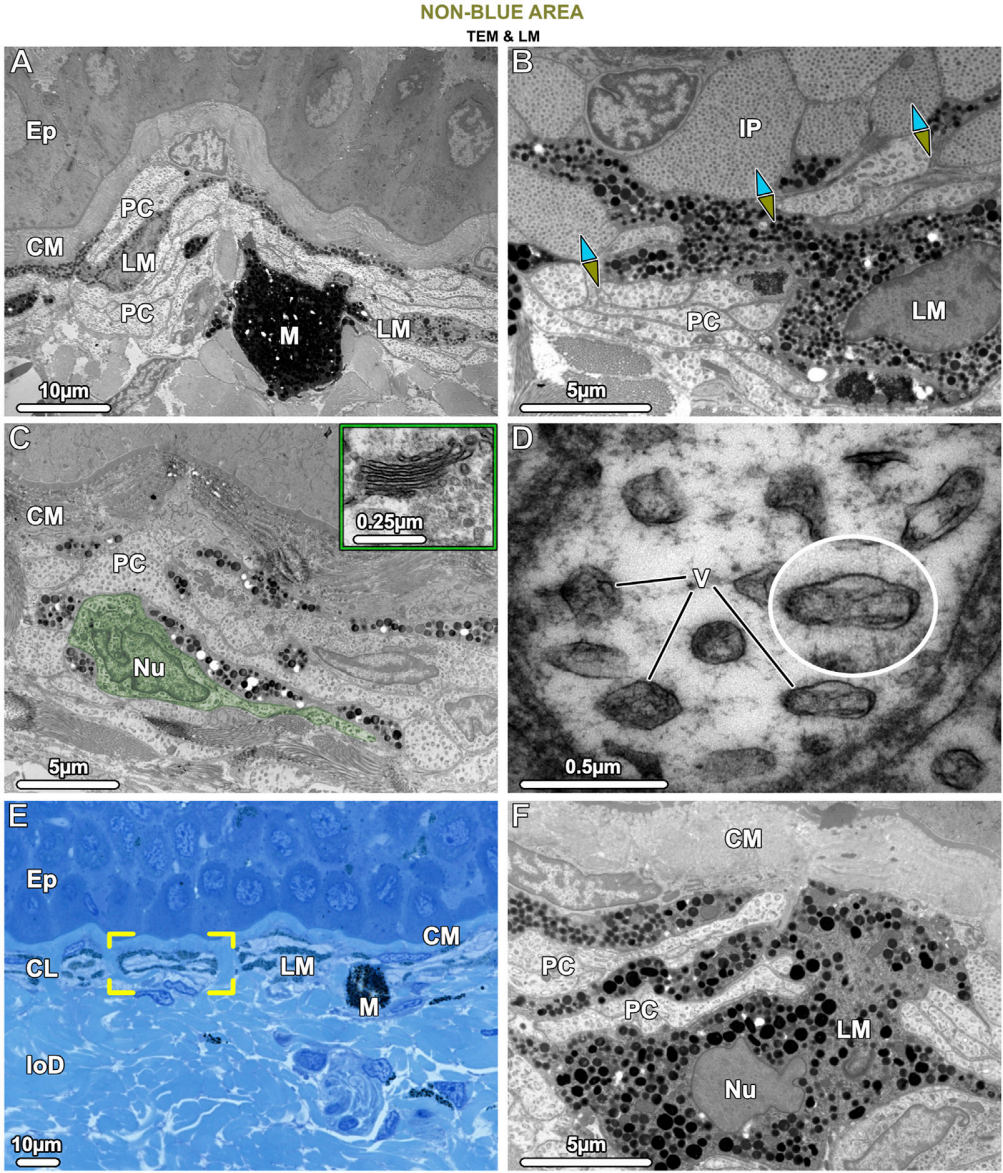
TEM **(A–D, F)** and LM **(E)**. Ultrastructural characteristics of the pale cells (PC) and the brown (LM) and black (M) melanophores in tissue of the non-blue area. **(A)** Pale cells and brown, light-pigmented melanophores (LM) are visible beneath the collagen mat (CM), the latter cell type having relatively loosely packed melanosomes. Both pale cells and brown melanophores have numerous slender processes arranged in alternating layers (also seen in **E, F**), with the melanophore being the most superficial. The comparatively rare black, dark-pigmented melanophores (M) are located immediately below this stratified layer, are round in shape, and densely packed with melanosomes. **(B)** The interface between blue and non-blue regions is delineated by iridophores (IP) and pale cells in contact, with the ultrastructural differences between the two cell types evident, especially in their iridosome/vesicle arrangements. The blue arrows point to the blue area, the green-brown arrows to the non-blue area. **(C)** In non-blue areas, the pale cells (PC, with one pale cell highlighted in green) have numerous thin, closely spaced processes and large vesicles. A Golgi apparatus near the nucleus (Nu) was usually not visible and could be observed only occasionally in the pale cells (inset). **(D)** Pale cell vesicles (V) have various shapes and sizes and contain fibrillar material but no crystal inclusions. The cytoskeleton is only weakly developed. **(E)** The brown melanophores (LM) exhibit long and horizontally aligned processes, in some cases even turning 180° back on themselves (brackets). **(F)** The processes of the brown melanophores (LM) are interleaved with those of the pale cells (PC), with the first processes directly beneath the mat of collagen (CM) and the others slotted between the pale cells. Ep = Epidermis; CL = Chromatophore layer of the upper dermis; loD = lower dermis.

In contrast to the blue areas, the dermal chromatophore layer of the non-blue areas of both juvenile and adult specimens was only 10–15 µm thick and consisted of brown, light-pigmented melanophores and pale cells (see below). Immediately below this layer, individual black (dark-pigmented), rounded melanophores with few short processes were observed (Figures 4B, D; Figure 8A), corresponding to the sparse black melanophores observed with stereomicroscopy (Figure 1C).

The cells we refer to as pale cells were only observed in the non-blue regions, inconspicuous in thick cryosections and visible for the first time only in semi-thin sections (Figures 4B, D). Pale cells had a similar gross morphology to iridophores and location in the skin’s chromatophore layer, but with notable differences. In TB sections, the cytoplasm of the pale cells stained similarly to iridophores from the blue regions, but TEM observations showed that they differed markedly from iridophores in both shape and subcellular architecture (Figure 8B). In comparison with iridophores, the pale cells had more slender processes (≤2.5 µm wide and 25 µm long) and contained larger vesicles with more variable shape and size (221.1 ± 40.18 nm in diameter) (Figure 10A). The intervesicular space (distance to nearest neighbors) was twice as large (121.32 ± 36.12 nm) as between iridosomes in iridophores (Figures 8C, D; Figure 10A). Additionally, although pale cell vesicles had a fibrillar content, we could not detect any vesicles with crystal-like inclusions or empty spaces to suggest crystal loss in sample preparation (Figure 8D). The cytoplasm of the pale cells comprised a lobulated nucleus and several mitochondria but, although we examined a great number of pale cells in TEM, we rarely found Golgi apparatuses surrounded by clustered vesicles, as in blue areas (Figure 8C). The cytoskeleton was weakly developed and comprised only a small number of filaments (8–10 nm in diameter), resulting in a more electron-translucent cytoplasm in pale cells than in iridophores (Figures 8B, D).

Similar to the association of melanophores and iridophores in blue regions, both melanophores and pale cells formed small structural units separated from each other by vertical collagen bundles (Figure 8E brackets). The brown melanophores (which we only observed in non-blue areas) had numerous thin processes (≤30 µm long) arranged in a horizontally layered architecture. These processes were interleaved with those of the pale cells with the first processes lying immediately below the sub-epidermal mat of collagen fibrils and the others sandwiched between the pale cells (Figures 8E, F). The brown melanophores contained round or ellipsoidal melanosomes that were loosely packed and less electron-dense than those of black melanophores (Figures 8A, F). Melanosomes with crystalline inclusions were found in neither brown nor black melanophores in the non-blue areas. The size of the melanosomes did not differ from that of the blue regions.

## Discussion

Despite the body of work on shark denticle hydrodynamics (e.g., Lang et al., 2012; Oeffner and Lauder, 2012), there is a shocking lack of information on the basic skin biology of sharks and rays: the scant information summarized in Meyer and Seegers (2012) more than a decade ago sadly remains the state of the art, with core concepts of tissue organization and patterning still undescribed. For example, our results apparently provide the first demonstration of brown melanophores in elasmobranchs, likely responsible for the huge diversity of brown and yellow skin mottling among shark and ray species (Last et al., 2016; Ebert et al., 2021). Moreover, our findings demonstrate important associations between different chromatophores (black and brown melanophores, pale cells and iridophores) in the skin of ribbontail stingrays that are key to color production, but also perhaps play a role in the ontogenetic development of distinct colors in different body regions (see below).

### Chromatophore units are key organs in skin color

**FIGURE 9:**
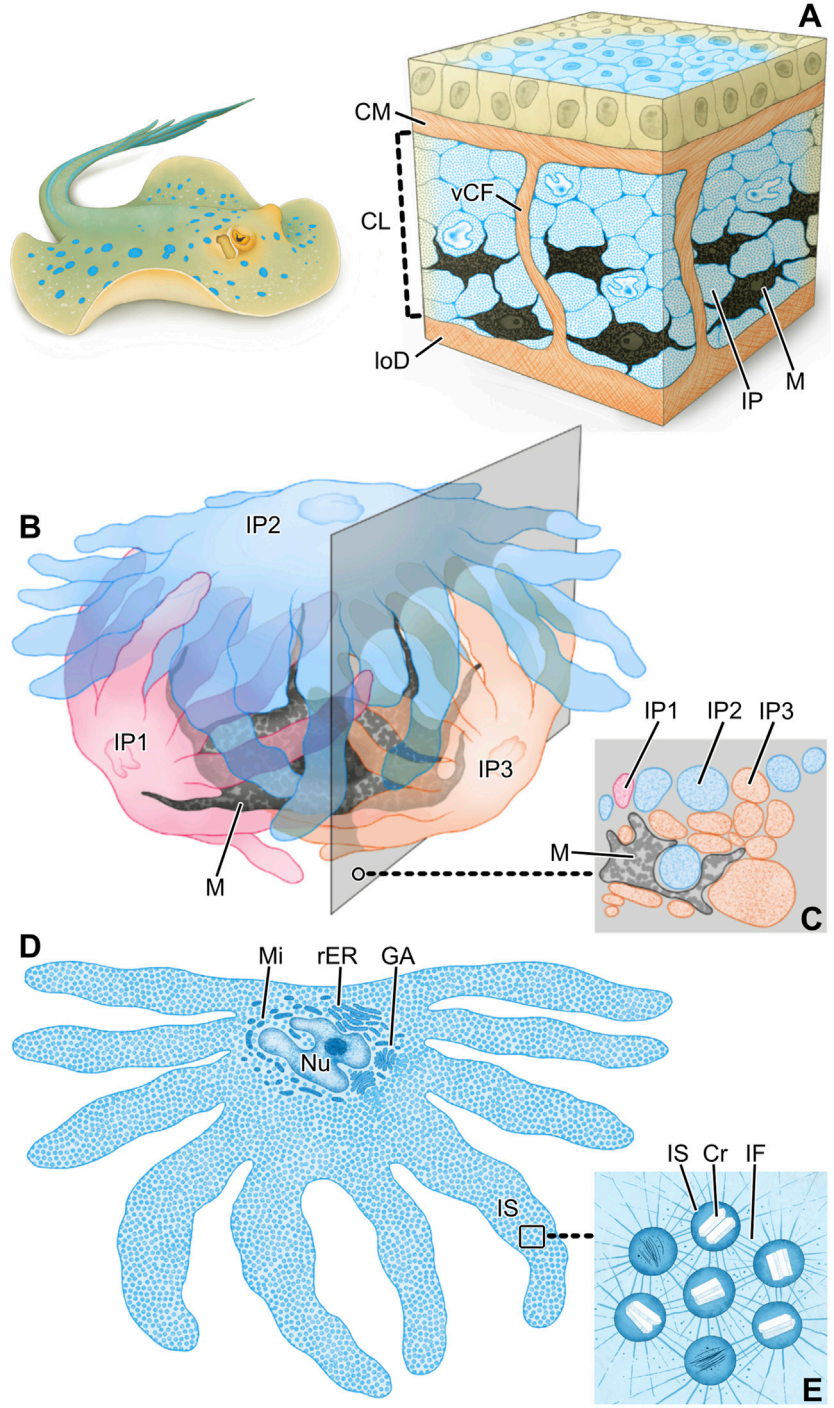
Schematics summarizing the hierarchical architecture of color-producing tissue in ribbontail stingray. In blue regions of the skin, the chromatophore layer sandwiched between the collagen mat (CM) and lower dermis (loD) **(A)** consists of densely arranged iridophores (IP) and melanophores (M) grouped together in chromatophore units and separated by vertical collagen bundles (vCF). Each chromatophore unit **(B)** involves a single melanophore (M) and multiple iridophores (IP), enfolding the black pigment melanophore with their numerous fingerlike extensions. Note how the section plane intersects portions of all three iridophores **(C)**. In slices through individual iridophores **(D)**, they are seen to be densely packed with iridosomes (IS), the cellular organelles such as the nucleus (Nu) and Golgi apparatus (GA), mitochondria (Mi) and rough endoplasmic reticulum (rER) consolidated to the cell’s central body. The boxed area is depicted at higher magnification in **(E)**. Iridosomes (IS) contain crystal platelets (Cr) and are kept in place relative to one another by a dense scaffold of intermediate filaments (IF).

The chromatophore units of ribbontail stingray provide a ‘packaging’ for the melanophore and iridophore association in the upper dermis of blue skin regions (Figure 4A; Figure 6E; see also tissue schematics in Figure 9, summarizing our findings): the iridophores produce the blue color, while the black melanophores are broadband absorbers, helping saturate the color (Figures 4A, C) (Surapaneni et al., 2024). Stingray chromatophore units are particularly discrete and spatially confined, sandwiched between upper and lower collagen layers and bounded laterally by vertical collagen bundles, which traverse the chromatophore layer like a building’s columns linking floor and ceiling (Figure 6E; Figure 9A). Within each chromatophore unit, the several iridophores surround their single melanophore, similar to multiple fists clenching the same black stone (Figure 4C; Figures 9B, C). Ribbontail stingray iridophores are strikingly odd chromatophores, in both morphology and crystallography. In vertebrates, iridophores are typically simple ovoids or polygons in cross section, lack cell processes, and are localized in the dermis above a layer of melanophores (Taylor, 1969; Taylor and Bagnara, 1972; Zhao et al., 2022). In contrast, ribbontail ray iridophores have numerous fingerlike projections (resembling sunstar echinoderms or ‘Buddha’s hand’ citrus fruits) and were observed in both the epidermis and dermis (Figure 5A inset; Figures 7A, C; Figure 9D see below). Of all described iridophores, these most closely resemble the reflector cells in the eyes of crustaceans, which also have finger-like projections, albeit less densely arranged than in the ribbontail stingray (Palmer et al., 2020; Shavit et al., 2023).

The tight tissue linkages and collagenous containment of chromatophore units likely provides structural integrity to the association of melanophore and iridophores, and thereby robustness and resilience to the skin color: this may explain how the blue color of ribbontail stingray can be recovered even after multiple freezing/thawing or dehydration/rehydration cycles (Surapaneni et al., 2024). The color of the blue spots also does not seem to vary with body movement of living stingrays (e.g., the curving of the wings during swimming; M. Dean and Ashlie McIvor, personal observations), suggesting the structure of the chromatophore unit (coupled with the non-iridescence of the color from iridosome arrangements; Surapaneni et al., 2024) helps to maintain a consistent optical signal in the environment, regardless of viewing angle or tissue deformation. The mechanical robustness of the color, therefore, makes the architecture of this system potentially interesting for the design of structural-colored flexible membranes (e.g., functional textiles).

Unlike the thickened collagen layers and bundles bounding them, chromatophore units (with their thin membranes and discontinuous internal architecture) likely play little role in skin integrity and material properties. This suggests a certain freedom in the evolution of coloration in elasmobranchs, as the tissue mechanisms underlying color production would not compete with constraints shaping tissue mechanics. In contrast, Bagnara et al. (2007) argued that the blue coloration of the ocellate torpedo ray (*Torpedo ocellata, syn. T. torpedo*) is due to blue wavelength reflection by a collagen layer in the dermis. Since the torpedo’s blue regions are localized to eyespots on its back, this would indicate that either the specific collagen organization differs in those areas (i.e., also affecting local mechanical properties) or that the collagen organization that reflects blue is ubiquitous in the integument, but is somehow blocked in non-blue regions from incident light, as in the zebra killifish *Fundulus heteroclitus*, where melanophores shield thick collagen bundles that produce blue skin color elsewhere (Junqueira et al., 1970). Although Bagnara et al. (2007) did not address this topic explicitly and no histological images exist for torpedo ray skin, neither option is well supported in Bagnara et al.‘s work: the collagen layer was not mentioned to differ between blue and non-blue regions and the tissue in both areas appears similar in the authors’ sketch (based on TEM and LM observations), with only sparse melanophores above the collagen layer. We therefore believe that collagen is likely not responsible for the blue coloration of electric rays and that the mechanism of color production should be revisited; following on from our work and that of Surapaneni et al. (2024), particular attention should be paid to detecting chromatophore units in electric rays.

### Iridophores migrate from the dermis to the epidermis

Epidermal iridophores in ribbontail stingrays had the same ultrastructural organization as those of the dermis, but were separated from each other by mucus-containing cells, not organized into tight chromatophore units (compare Figures 7A, C). The presence of iridophores in the epidermis is intriguing and, to the best of our knowledge, has only been shown in an invertebrate, the marine nudibranch *Flabellina iodine* (Dearden et al., 2018). In vertebrates, the only chromatophores typically found in the epidermis are melanophores, whereas the dermis can contain all three subclasses of chromatophores (Taylor and Bagnara, 1972). In the fire salamander, however, xanthophores migrate from the dermis into the epidermis during metamorphosis after penetrating the basement membrane, resulting in the adult skin pattern of yellow spots or stripes on black skin. The xanthophores remain loosely anchored to their surroundings even after settling in the epidermis, not forming desmosomes with their neighboring cells (Pederzoli et al., 2003).

Our data suggest a similar scenario for the ribbontail ray, where in young animals, we observed individual iridophores that appeared to be migrating, having detached from the chromatophore layer and penetrated the overlying collagen fibril mat and basement membrane, with those iridophores already established in the epidermis lacking strong adhesion to their neighboring cells (Figures 7D–F). Whether there is an optics-related role of this migration is unclear, but investigation of this process will surely reveal important cellular associations in the function of stingray iridophores (e.g., whether certain chromatophores attract or repel them; see below).

Despite the suggestion of ontogenetic stages of iridophore development (e.g., migration between tissue layers), we observed a consistent iridophore morphology between juvenile (e.g., Figure 7C) and adult individuals (e.g., Figure 5A). Moreover, the internal colloidal system of iridosome vesicles was maintained, with its short and consistent cytoplasmic distance between organelles, indicating that the overall light-reflecting ultrastructure of the cells—and the blue color it produces (see spectroscopy data in Surapaneni et al., 2024)— also do not change with development. This ontogenetically stable pattern differs markedly from that of lizards, in which juveniles have a conspicuous blue tail coloration (to distract predators from their vital body organs), which is lost in adults to be replaced by a cryptic light brown color. This color change is due to an increasing number of iridophore layers, maturation and realignment of the crystals coupled with pigment deposition in the overlying xanthophores of adult lizards (Kuriyama et al., 2016; Zhang et al., 2023). In stingrays, the structural consistency in iridophores across ontogeny, however, does not preclude them (or the color they produce) from playing different roles in the species’ ecology at different life stages since juveniles and adults tend to occupy different habitats (Surapaneni et al., 2024).

### Melanophores may determine the fate of iridophore precursor cells

In vertebrates, chromatophores all derive from neural crest (Figon et al., 2021) explaining the existence of several heterogeneous cell types: mixed chromatophores with at least two types of pigment organelles or, less commonly, mosaic organelles containing different color-producing materials simultaneously (Taylor, 1971; Bagnara et al., 1979). In ribbontail stingray, we observed the latter: melanophores in the blue regions containing a small number of melanosomes with rectangular crystals. The crystal composition remains unclear, however, as they did not turn dark with phosphotungstic acid, indicating that they do not have a high nitrogen content (Sternberg, 1970) (Figures 6B–D). Mosaic organelles containing crystals and melanin have never been seen in skin, but have been observed in other animal tissues, mostly in relation to a silvery reflective quality: in the tapetum lucidum of the stingray *Dasyatis sabina* (Arnott et al., 1970), the liver of the frog *Pachymedusa dacnicolor,* and the iris of doves (Bagnara et al., 1979). Based on the optical performance of structurally analogous artificial polydopamine-polystyrene core-shell particles (Kawamura et al., 2016; Kohri et al., 2017) and depending on the refractive index differences between the crystalline structures and melanin, these mosaic organelles theoretically have the ability to produce a variety of structural colors. However, their apparent sparseness in ribbontail ray skin indicates that they would likely only play a minor role, if they were indeed involved in color production.

**FIGURE 10:**
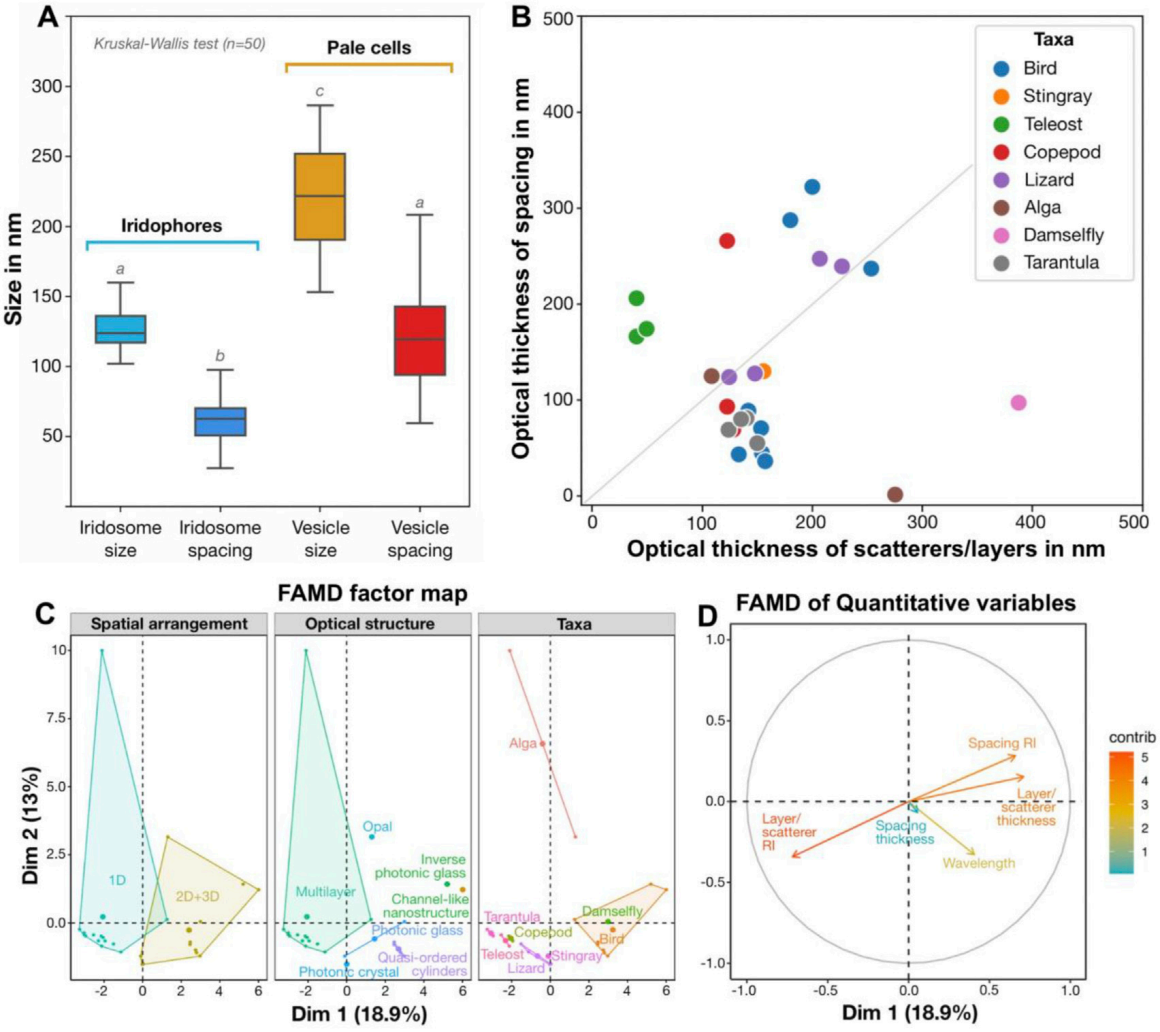
Quantitative results in context, comparing **(A)** nanostructuring in blue and non-blue regions and **(B–D)** the architectures behind diverse blue structural colors **(B–D)**. **(A)** Quantitative differences (Kruskal-Wallis and Dunn *post hoc* tests, n = 50; marked as lowercase letters) in the size and spacing of iridosomes (crystal-containing vesicles) in iridophores in the blue skin region and empty vesicles in pale cells in the non-blue skin region. **(B)** Comparison of optical thicknesses of scatterers/layers and their spacing in different taxa, with some taxa demonstrating deviation from the theoretical case of an ideal multilayered material with high reflectivity (grey line: optical thicknesses of layers and spacing are equal to a quarter of the wavelength). **(C, D)** Factor Analysis of Mixed Data (FAMD) reveals qualitative variables (e.g., arrangement, structure and taxa) that drive variation among diverse examples of natural blue structural colors **(C)**, as well as highlighting probable co-occurrence of several quantitative variables (e.g., refractive index of the space between layers and layer thickness) along dimensions 1 and 2 (Dim1 and Dim2, respectively) **(D)**. (see references in Supplementary Table S1).

Whether in blue or non-blue skin regions, stingray melanophores showed close association with iridophores and iridophore-like pale cells, respectively. Similar to the chromatophore units of the blue regions, the pale cells and light-pigmented (brown) melanophores of the non-blue regions were located in the upper dermis, entwined and bounded by collagen fibers (Figure 8E). Also, pale cells had a similar general appearance to iridophores, with numerous cell processes, several mitochondria, cytoplasm dominated by nanovesicles, and lobed nuclei (Figures 8B–D). We therefore posit that pale cells and iridophores share the same progenitor cell, but are triggered during development to spatially differentiate into separate cell types with ultrastructural differences relevant to color production. In comparison to the iridophores, the pale cells showed a poorly developed cytoskeleton, a negligible Golgi apparatus, thinner cell processes and larger vesicles (221.1 ± 40.18 nm in diameter), which had a fibrillar content (like iridosomes) but showed no ultrastructural evidence of crystal formation (Figures 8A–D). In addition, the intervesicular distances were twice as large and more variable than in iridophores (Figure 10A). These differences relative to iridophores therefore reinforce the hypothesis that the precise spacing and resultant quasi-order of vesicles and their crystals are the bases of the coherent scattering of blue light in ribbontail stingray chromatophore units (Surapaneni et al., 2024). The factors driving the spatial differentiation of pale cells and iridophores from their common progenitor require attention, especially as a sharp distinction between blue and non-blue areas is vital for creating high chromatic contrast (important for how this species is perceived in its environment; Marshall et al., 2019).

We propose that the presence and type of melanophores may be driving factors in skin patterning in ribbontail stingray, based on the cellular interactions we observed in blue and non-blue areas and on signaling pathways that determine skin coloration in other vertebrates. In zebrafish (*Danio rerio*) stripe formation, for example, iridophore precursor cells develop first in larvae, with their fates decided by interactions among different chromatophore types. In later development, a specific microenvironment (absence or presence of melanophores) triggers the differentiation of iridophores into two subtypes of light-reflecting cells, which differ in architecture as well as cytoplasmic spacing of the crystals, eventually contributing to either the blue stripe or the yellow interstripe (Hirata et al., 2003; Frohnhöfer et al., 2013; Mahalwar et al., 2014; Gur et al., 2020).

Similarly, our observations of cell covariation suggest a progenitor cell’s local environment could control the development of light-reflecting organelles/cells in stingray skin, perhaps linked to the presence/absence of specific melanophore types. The skin patterns we observed in stingrays could be achieved, for example, if light-pigmented melanophores could suppress crystal formation in progenitor cells in non-blue areas (Figure 1C; Figures 8E, F), whereas crystal formation would occur where such melanophores were absent, namely, in the prospective blue areas. The intimate relationship of black melanophores and iridophores in chromatophore units further supports this proposed communication between chromatophore types (e.g., Figure 7D). The ribbontail ray has live birth, with blue spots already formed in hatchlings (Ashlie McIvor, personal communication), meaning that the ultrastructural differences between blue and non-blue regions are established during embryonic development. This, unfortunately, challenges experimental efforts to unravel the mechanisms driving local cell differentiation and crystal formation; embryonic specimens, experimental treatments (e.g., chemically/hormonally disrupting melanophores; Visconti and Castrucci, 1993), and investigations of skin tissue changes following damage would offer inroads for understanding this phenomenon.

### Skin color is mediated by small crystals and intermediate filaments

From a physical-optical point of view, the material, size and spacing of the crystals in ribbontail stingray iridosomes (Figure 5C; Figure 10A) are crucial for color production. Our recent work (Surapaneni et al., 2024) confirmed that the light-reflecting crystals in iridosomes are composed of anhydrous beta-guanine, a common natural material with high nitrogen content and refractive index (n = 1.83) involved in the structural coloration. From plankton to vertebrates, the guanine crystals involved in color production form through a sequence of morphologically well-defined stages (Bagnara, 1983; Figon et al., 2021; Pinsk et al., 2022). Our observations of ribbontail stingray tissues suggest a mechanism of crystal development very similar to zebrafish, the white widow spider *Latrodectus pallidus,* and the scallop *Pecten maximus*, where (i) crystals form near the Golgi apparatus (Figure 5D) (Wagner et al., 2022), (ii) small Golgi vesicles fuse with early iridosomes (Figure 5D) (Wagner et al., 2022; Wagner et al., 2023) and (iii) a pre-assembled scaffold of amyloid fibrils inside iridosomes drives crystal growth (Figure 5E) (Eyal et al., 2022; Wagner, et al., 2023). The development of iridosomes suggested by our data is also strikingly similar to the well-documented morphogenesis of melanosomes (Hurbain et al., 2008; Wagner et al., 2023), further strengthening the idea that stingray melanophores and iridophores have a common neural crest progenitor cell, and melanosomes and iridosomes share a common endosomal origin (Figon et al., 2021).

In the ribbontail stingray, we observed some larger crystals with an elongated shape (∼300 nm long), but the vast majority remained exceptionally small (∼85 nm) in comparison with most other known vertebrate iridophores (Figure 5C; Figures 10A, B), suggesting that the process of crystal development is truncated in stingrays. The mechanism that dictates crystal shape is still unclear, but recent studies on other species have ruled out the iridosome membrane as the limiting factor (Wagner et al., 2022; Wagner et al., 2023). In the skin of most fishes, amphibians, reptiles, and some invertebrate species, guanine crystals are large (several µm long) and in a multilayer arrangement, with individual platelets stacked on top of each other, separated by a cytoplasmic layer with a much lower refractive index (*n* = 1.33) than that of the crystals (*n* = 1.83; Taylor and Bagnara, 1972; Goda et al., 1994; Kottler et al., 2014; see additional references in Supplementary Table S1). Such a multilayered arrangement of crystalline sheets produces visible colors when the optical thicknesses (product of refractive index, *n* and thickness, *d*) of the crystal sheets and of the cytoplasmic layers separating them are of a comparable nanometer-scale to the wavelength of light. In copepods and some teleosts, the reflected color is primarily determined by the spacing between crystal layers, whereas the crystal layer thickness is constant (Denton and Land, 1971; Amiri and Shaheen, 2012; Sun et al., 2013; Gur et al., 2015b; Gur et al., 2017; Gur et al., 2020). Similarly, in a 3D photonic glass, such as the iridophore of the ribbontail stingray, the cytoplasmic spacing between crystal-bearing iridosomes determines the resonant wavelengths coherently scattered from the colloidal system (Magkiriadou et al., 2014; Surapaneni et al., 2024). Outside of stingrays, such a uniform and stable arrangement of small crystalline organelles has so far only been reported in the reflector cells of crustacean eyes (Palmer et al., 2020; Shavit et al., 2023).

In our previous study (Surapaneni et al., 2024), we demonstrated a constant distance between crystals, and here we provide evidence of a well-developed cytoskeleton between iridosomes, suggesting a tensegrity network that maintains the necessary stable colloidal arrangement of short guanine crystals to produce a structural blue (Figures 5F, G; Figure 9E). Based on cytoplasmic spacing and the crystals not completely filling iridosomes (Figure 5C; Figure 9E), we measured a distance of ∼100 nm between crystals, similar to the distance between platelets in other vertebrate structural blues (Figure 10B). In fact, our metadata FAMD analysis shows that variation among reported natural examples of blue structural color is largely driven by architectural and material variables that determine optical thickness (those loading heavily on Dim1 and Dim2: e.g., the material, spacing and shape of scatterers and/or the matrix between them; Figures 10C, D). Similarly, the observed variation among taxa (e.g., birds vs. fishes), among optical structures producing color (e.g., multilayers vs. quasi-ordered arrays) and their spatial arrangements (e.g., 1D or 2D/3D) is distributed largely over Dim 1 with groups represented in relatively discrete clumps (barely, if at all, overlapping; Figure 10C). This clumping of related groups suggests the factors shaping optical thickness are tied to phylogeny, tissue architecture, and optical performance; however, broader versions of this survey would help pinpoint fundamental factors and selective pressures driving structural color evolution across a huge phylogenetic scope.

Although the components of the cytoskeleton that provide constant spacing between crystals are crucial to the control and production of structural color in vertebrate iridophores, they have received little attention in the literature, likely because they are more challenging to image than the crystals themselves. In fact, despite several studies having observed fibrils in light-reflecting cells, few have even commented on their presence (Taylor, 1969; Rohrlich and Porter, 1972; Rohrlich, 1974). In the lizard *Anolis carolinensis* and two teleost species, *Holocentrus adscensonis* and *Carassius auratus*, iridophores contain two classes of filaments: thick intermediate filaments (10 nm diameter) and thin actin filaments (6.5 nm diameter) forming an elaborate network in the cytoplasm. The thin filaments allow for crystal movement and cell shape changes, while the thick filaments act as a cytoskeleton, holding crystals precisely in place (Rohrlich and Porter, 1972; Rohrlich, 1974). Similarly, the dense cytoskeletal scaffold in ribbontail stingray iridophores are likely intermediate filaments, based on their diameter (Figures 5F, G; Figure 9E). In other animals, thick intermediate filaments are known to form remarkably flexible and viscoelastic interconnected networks (Fudge et al., 2003; Böni et al., 2018), promoting important dynamic behaviors to intracellular components of skin when hydrated. We therefore posit that the hydration-sensitive mechanical properties (e.g., extensibility, stiffness) of the intermediate filament scaffold in stingray iridophores are key factors for maintaining the necessary inter-crystal spacing to produce blue structural color. This is similar to the hydration-induced structural color of red algae (Chandler et al., 2015) and supports our previous observation that rehydration returns the bright blue structural color of dried ribbontail stingray skin (Surapaneni et al., 2024). Consequently, these dynamic mechanical properties of the intermediate filament matrix in ribbontail stingray iridophores also present an excellent model for designing tunable man-made photonic glasses.

## Conclusion

This study describes how distinct local variation of two chromatophore types (iridophores, melanophores) is responsible for the electric blue spots of the ribbontail stingray. We identify a new type of structurally-complex iridophore, involving crystalline iridosomes arranged in a stable colloidal pattern suspended by an intricate cytoskeleton. Iridophores interact in distinct organs (chromatophore units) in the dermis of blue regions, enveloping single melanophores: our data show a key link between the iridophore ultrastructural features necessary for blue color production (e.g., iridosome spacing and crystallinity) and the presence of dark melanophores as broadband-absorbers, further suggesting that chromatophore co-association is key in this system and that variation in this relationship may explain individual color/pattern variation in nature (e.g., relating to important ecological factors like mate choice and camouflage). We show the architecture of the chromatophore layer and the ultrastructure of iridophores remains unchanged during ontogeny, indicating that the blue skin color is consistent and likely ecologically important throughout the animal’s life. Future work investigating the migration of cells (e.g., iridophore movement from dermis to epidermis) and differentiation of cell types (e.g., from iridophore precursor to iridophore or pale cells) will clarify what drives chromatophore interactions in specific regions. There is considerable interest in industry to mimic nature’s angle-independent structural colors (Iwata et al., 2017; Xue et al., 2022), since structural colors, unlike pigments, do not fade with time. Understanding the nature of tissue scaffoldings for structural color production and the control they exert on shape and spacing of scattering elements could improve control in biotemplating efforts (e.g., of guanine crystals), a target for improving performance in diverse advanced synthesized materials, from tunable reflectors and dyes to optoelectronic devices and organic semiconductors.

## Data Availability

The data presented in the study are available at the following link: https://doi.org/10.6084/m9.figshare.26125378.v1.
