# Supplementary material for: Intermediate filaments spatially organize intracellular nanostructures to produce the bright structural blue of ribbontail stingrays across ontogeny
Source: Front Cell Dev Biol. 2024 Jul 10;12:1393237. doi: 10.3389/fcell.2024.1393237 (PMC11266302; doi:10.3389/fcell.2024.1393237)
Supplement: Supplementary file 1 [file DataSheet1.PDF]

Table S1. Collected literature examples of morphological and optical features from natural structural “blues” (388-490 nm) from diverse eukaryotic groups (vertebrates, invertebrates and algae).

Taxa	Species	Optical structure	Angle dep.	λ (nm)	Material		Refractive index		Thickness		Shape		Mechanism	Literature
					Core	Spacing	Core	Spacing	Core	Spacing	Core	Spacing		
Bird	*Cotinga cotinga*	Inverse Photonic glass	No	462	Air cavity	Keratin	1	1.58	180	182	Spherical	Matrix	Coherent scattering	Noh et al., 2010
Bird	*Irena puella*	Channel-like nano-structure	No	478	Air channel	Keratin	1	1.58	200	204	Channel	Matrix	Coherent scattering	Noh et al., 2010
Bird	*Neodrepanis coruscans*	Quasi ordered array	No	417	Collagen fiber	Muco-saccharide	1.55	1.35	85.9	32.1	Cylindrical	Matrix	Coherent scattering	Prum et al., 1999
Bird	*Philepitta castanea*	Quasi ordered array	No	483	Collagen fiber	Muco-saccharide	1.55	1.35	91.5	65.8	Cylindrical	Matrix	Coherent scattering	Prum et al., 1999
Bird	*Neodrepanis hypoxantha* 1	Quasi ordered array	No	465	Collagen fiber	Muco-saccharide	1.55	1.35	99	52.2	Cylindrical	Matrix	Coherent scattering	Prum et al., 1999
Bird	*Neodrepanis coruscans*	Quasi ordered array	No	517	Collagen fiber	Muco-saccharide	1.55	1.35	99.5	33.1	Cylindrical	Matrix	Coherent scattering	Prum et al., 1999
Bird	*Neodrepanis hypoxantha 2*	Quasi ordered array	No	403	Collagen fiber	Muco-saccharide	1.55	1.35	101.4	26.8	Cylindrical	Matrix	Coherent scattering	Prum et al., 1999

Stingray	*Taeniura lymma*	Photonic glass	No	450	Guanine	Cytoplasm	1.83	1.3	85	100	Cuboidal	Matrix	Coherent scattering	Surapaneni et al., 2024
Teleost	*Paracheirodon innesi* 1	Multilayer	Yes	490	Guanine	Cytoplasm	1.83	1.33	22	155	Cuboidal	Matrix	Multilayer interference	Gur et al., 2015a
Teleost	*Paracheirodon innesi* 2	Multilayer	Yes	400	Guanine	Cytoplasm	1.83	1.33	22	125	Cuboidal	Matrix	Multilayer interference	Gur et al., 2015a
Teleost	*Danio rerio*	Multilayer	Yes	450	Guanine	Cytoplasm	1.83	1.33	27	131	Cuboidal	Matrix	Multilayer interference	Gur et al., 2020
Copepod	*Sapphirina metallina* 1	Multilayer	Yes	430	Guanine	Cytoplasm	1.83	1.33	67	200	Perfect hexagon	Matrix	Multilayer interference	Gur et al. 2015b
Copepod	*Sapphirina metallina* 2	Multilayer	Yes	485	Guanine	Cytoplasm	1.83	1.33	67	70	Perfect hexagon	Matrix	Multilayer interference	Gur et al. 2015b
Copepod	*Copilia mirabilis*	Multilayer	Yes	460	Guanine	Cytoplasm	1.83	1.33	70	52	Perfect hexagon	Matrix	Multilayer interference	Gur et al. 2015b
Reptile	*Furcifer pardalis* 1	Photonic crystal	No	480	Guanine	Cytoplasm	1.83	1.33	113	186	Cuboidal	Matrix	Coherent scattering	Teyssier et al., 2015
Reptile	*Furcifer pardalis* 2	Photonic crystal	No	480	Guanine	Cytoplasm	1.83	1.33	124	180	Cuboidal	Matrix	Coherent scattering	Teyssier et al., 2015
Lizard	*Phelsuma grandis*	Multilayer	No	480	Guanine	Cytoplasm	1.83	1.33	80.8	96	Cuboidal	Matrix	Multilayer interference	Saenko et al., 2013
Lizard	*Phelsuma klemmeri*	Multilayer	No	410	Guanine	Cytoplasm	1.83	1.33	68	93.2	Cuboidal	Matrix	Multilayer interference	Saenko et al., 2013
Alga	*Chondrus crispus*	Multilayer	Yes	400	Lamellae	Interlamellar region	1.55	1.47	70	85	Sheet	Sheet	Multilayer interference	Chandler et al., 2015

Alga	*Cystoseira tamariscifolia*	Opal	Yes	440	Lipid	Cytoplasm	1.48	1.35	186	1	Spherical	Matrix	Coherent scattering	Lopez-Garcia et al., 2018
Bird	*Phasianus colchicus*	Multilayer	Yes	480	Melano-some	Keratin	1.83	1.58	138.54	150.06	Cylindrical	Matrix	Multilayer interference	Jeon et al., 2023
Damselfly	*Enallagma civile*	Photonic glass	No	475	Protein	Cytoplasm	1.55	1.35	250	72	Spherical	Matrix	Coherent scattering	Prum et al., 2004a
Tarantula	*Omothymus violaceopes*	Multilayer	Yes	424	Chitin	Air	1.63	1	92	55	Cuboidal	Matrix	Multilayer interference	Hsiung et al., 2015
Tarantula	*Ephebopus cyanognathus*	Multilayer	Yes	388	Chitin	Air	1.63	1	76	69	Cuboidal	Matrix	Multilayer interference	Hsiung et al., 2015
Tarantula	*Caribena laeta*	Multilayer	Yes	444	Chitin	Air	1.63	1	86	81	Cuboidal	Matrix	Multilayer interference	Hsiung et al., 2015
Tarantula	*Poecilotheria metallica*	Multilayer	Yes	432	Chitin	Air	1.63	1	83	80	Cuboidal	Matrix	Multilayer interference	Hsiung et al., 2015
